# ILC3-derived acetylcholine promotes protease-driven allergic lung pathology

**DOI:** 10.1016/j.jaci.2020.10.038

**Published:** 2021-04

**Authors:** Matthew Darby, Luke B. Roberts, Claire Mackowiak, Alisha Chetty, Sasha Tinelli, Corinna Schnoeller, Valerie Quesniaux, Sylvie Berrard, Dieudonnée Togbe, Murray E. Selkirk, Bernhard Ryffel, William G.C. Horsnell

**Affiliations:** a Wellcome Centre for Infectious Diseases Research in Africa, Institute of Infectious Disease and Molecular Medicine, Department of Pathology, Division of Immunology, University of Cape Town, Cape Town, South Africa; b Department of Life Sciences, Imperial College London, London, United Kingdom; c School of Immunology and Microbial Sciences, King’s College London, London, United Kingdom; d INEM UMR7355 Experimental and Molecular Immunology and Neurogenetics, CNRS and University of Orleans, Orleans, France; e NeuroDiderot, Inserm, Université Paris, Paris, France; f Institute of Microbiology and Infection, College of Medical and Dental Sciences, University of Birmingham, Birmingham, United Kingdom

To the Editor:

Initiation of allergic airway pathology often depends on the protease activity of the inhaled allergen. Previous studies have shown that IL-17–driven neutrophil and eosinophil responses can promote allergic pathology^1^ and are associated with an initial epithelial release of IL-23, which is accepted as important in promoting T_H_17 and group 3 innate lymphoid cell (ILC3) responses. ILC3s are a developmentally and phenotypically diverse innate lymphoid cell (ILC) subset that includes natural killer (NK) cell receptor (NCR)-positive and NCR-negative populations (NCR^+^ ILC3s and NCR^–^ ILC3s) as well as CCR6^+^ lymphoid tissue inducer cells. However, all ILC3s are defined by expression of transcript variant 2 of *RORC*, encoding retinoid-related orphan receptor γt (RoRγt) and production of IL-17 and IL-22.^1^^,^^2^ Work carried out in murine models has resulted in ILC3s emerging as critical regulators of infectious^2^ and noninfectious^1^ pulmonary diseases despite ILC3s representing only a minor lung immune cell population in mice. In contrast, ILC3s are the major ILC population in the human lung.^3^ Importantly, ILC3-associated preclinical phenotypes are reflective of observations in humans.^2^^,^^4^ However, our understanding of the molecular machinery enabling ILC3s to exact their influence on lung immunity is incomplete.

In this study, we have demonstrated that expansion of lung ILC3s occurs in response to the protease papain and that these cells promote an IL-17–associated lung pathology. Critically, we have shown that induction of papain-driven pathology is strongly associated with ILC3 synthesis of acetylcholine (ACh). We have previously identified ACh responsiveness by immune cells in the lung as being important for CD4^+^ T-cell–driven adaptive immunity to *Nippostrongylus brasiliensis* infection.^5^ Here, we have extended this insight by demonstrating that ACh from lineage-negative (Lin^–^) CD127^+^ lymphocytes expressing RoRγt is instrumental for promoting protease induction of allergic inflammation. This identifies a new paradigm for how ILC3s and ACh contribute to early promotion of allergic pathology that is distinct from our traditional understanding of ACh-driven neuromuscular interactions causing allergic pulmonary airway resistance.

We have identified association of ILC3s with protease-induced lung pathology following acute papain challenge of wild-type (WT) C57BL/6 mice. Papain challenge increased lung concentrations of IL-13, IL-17A, IL-22, and IL-23 in comparison with the concentrations in saline-challenged mice **(**Fig 1, *A*). IL-17– and IL-23–promoted pathology suggests a RoRγt-driven inflammation. Papain challenge of RoRγt–green fluorescent protein (GFP) reporter mice demonstrated a significant expansion of the numbers of ILC3s (Lin^–^CD45^+^CD127^+^ICOS^–^RoRγt-GFP^+^), group 2 ILCs (ILC2s) (Lin^–^CD45^+^CD127^+^ICOS^+^RoRγt-GFP^–^), and CD3^+^CD4^+^ RoRγt-GFP^+^ T cells relative to the numbers in saline-treated controls (Fig 1, *B* and see Fig E1 in this article's Online Repository at www.jacionline.org). Restimulation and intracellular cytokine capture of lung CD4^+^ T cells from papain-challenged mice detected increased levels of IL-5 and IL-13 but not IL-17 when compared with the levels in saline-treated controls (Fig 1, *C*). However, in CD45^+^CD3^–^Lin^–^ cells, in addition to increased levels of IL-5 and IL-13, a raised IL-17 level was detected in papain-challenged mice when compared with the levels in saline-treated controls (Fig 1, *C*). Moreover, anti-CD3 depletion of T cells did not protect against and in fact promoted papain-driven pathology (see Fig E2 in this article's Online Repository at www.jacionline.org). These findings support IL-17–driven pathology as being independent of RoRγt^+^ T_H_17 T-cell IL-17 production.

**Fig 1:**
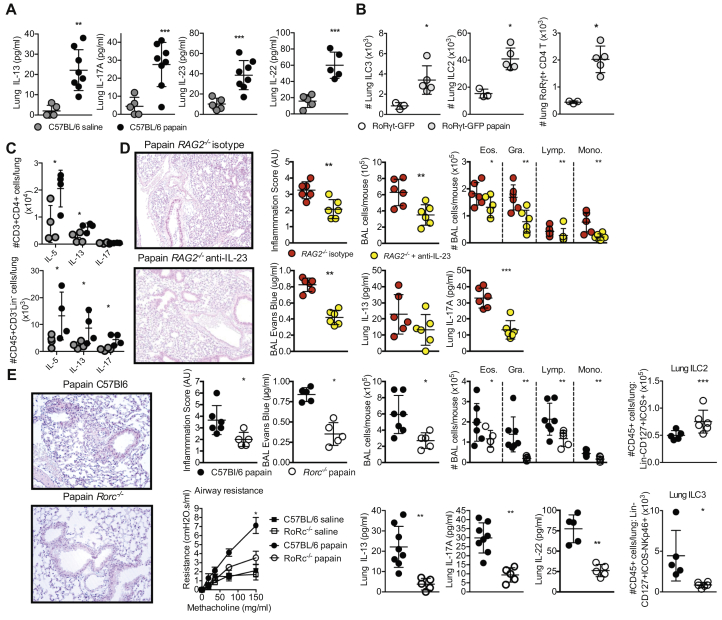
RoRγt -expressing cells drive protease-induced airway inflammation. **A,** Detection of pulmonary IL-13, IL-17A, IL-22, and IL-23 by ELISA following acute papain challenge. **B,** ILC3s (Lin^–^CD45^+^CD127^+^ICOS^–^RoRγt-GFP^+^), ILC2s (Lin^–^CD45^+^CD127^+^ICOS^+^RoRγt-GFP^–^), and T_H_17 T cells (CD3^+^CD4^+^RoRγt-GFP^+^) were detected in the lung by flow cytometry by using RoRγt-GFP reporter mice following acute papain challenge. **C,** Phorbol myristate acetate restimulation of lung cells potentiated production of IL-13 but not IL-17 by CD4^+^ T cells in papain-treated mice, as measured by flow cytometry. But CD45^+^CD3^–^Lin^–^ cells did show IL-17 production following phorbol myristate acetate restimulation. **D,** Representative hematoxylin and eosin staining of lung sections, pathology scoring, and quantification of airway epithelial integrity by using EB following papain challenge of *RAG2*^–/–^– and IL-23–depleted *RAG2*^–/–^ mice. Airway cellular infiltration and detection of pulmonary IL-13 and IL-17A by ELISA is also shown. **E,** Representative hematoxylin and eosin staining of lung sections, pathology scoring, and quantification of airway epithelial integrity by using EB following papain challenge of C57BL/6 and *Rorc*^–/–^ mice. Airway resistance to methacholine challenge following papain challenge in *Rorc*^–/–^ mice. Airway cellular infiltration in *Rorc*^–/–^ mice. Detection of pulmonary IL-13 and IL-17A by ELISA in papain-challenged *Rorc*^–/–^ mice. Detection of ILC2s (Lin^–^CD45^+^CD127^+^ICOS^+^) and NCR^+^ ILC3s (Lin^–^CD45^+^CD127^+^ICOS^–^NKp46^+^) in the lung by flow cytometry. Data are representative of 2 or 3 equivalent experiments; n = 3 to 9 mice/group. All data are a comparison of unchallenged and saline-treated or papain-challenged C57BL/6 background mice. Values represent means ± SDs; ∗*P* < .05; ∗∗*P* < .01; ∗∗∗*P* < .001. *BAL*, Bronchoalveolar lavage fluid; *Eos.*, eosinophil; *Gra.*, granulocyte; *Lymp*, lymphocyte; *Mono.*, monocyte.

To test a requirement for any T-cell (and B-cell) contribution to IL-23/IL-17–promoted pathology, we challenged *RAG2*^–/–^ mice with papain in the presence or absence of an IL-23–neutralizing mAb (anti–IL-23) (Fig 1, *D*). Decreased lung inflammation in IL-23–depleted *RAG2*^–/–^ mice was revealed by histologic analysis as well as by reduced detection of Evans blue (EB) leakage into bronchoalveolar lavage fluid (BALF) (Fig 1, *D*) when compared with that in isotype-treated *RAG2*^–/–^ mice. BALF immune cell infiltration was reduced in all immune cell populations, and tissue levels of IL-17A (but not IL-13) were lower in the IL-23–depleted *RAG2*^–/–^ mice (Fig 1, *D*). This abrogation of papain-induced allergic inflammation in anti–IL-23–treated *RAG2*^–/–^ mice supports ILC3s as being a key contributing lymphoid cell population driving protease-mediated lung inflammation.

To further characterize the input of ILC3s to allergic airway pathology, we compared pulmonary responses to papain in WT and *Rorc*^–/–^ mice (Fig 1, *E*). *Rorc*^–/–^ mice did not show significant baseline differences from C57Bl/6 mice in terms of BALF cell composition (see Fig E3 in this article's Online Repository at www.jacionline.org), but histologic analysis of lung sections revealed decreased inflammation and detection of EB leakage into the BALF in *Rorc*^–/–^ mice challenged with papain versus in WT mice (Fig 1, *E*). Total BALF immune cell infiltration was also reduced for all immune cell populations (Fig 1, *E*). Moreover, detection of both IL-13 and IL-17A, as well as IL-22, was reduced in *Rorc*^–/–^ mice (Fig 1, *E*). In agreement with findings by others^6^ *Rorc*^–/–^ mice had expanded numbers of ILC2s (Lin^–^CD45^+^CD127^+^ICOS^+^) when compared with the numbers in WT mice (Fig 1, *E*). However, as expected, detection of ILC3 subsets such as NCR^+^ ILC3s (Lin^–^CD45^+^CD127^+^ICOS^–^NKp46^+^) in *Rorc*^–/–^ mice was acutely reduced as opposed to in WT mice; the small number of cells detected were most likely to be non-NK, non–RoRγt-expressing group 1 ILCs (ILC1s). This body of work identifies a previously unappreciated, T-cell–independent role for IL-23–responsive ILC3s in contributing to the onset of papain-driven lung pathology.

An additional striking feature of these results was protection from cholinergic-promoted airway resistance during papain challenge in the absence of *Rorc* expression (Fig 1, *E*). Lymphocytes are important responders to, and sources of, neurotransmitters. For example, the ILC2 response to the neurotransmitter neuromedin U is critical for inducing type 2 immunity,^7^ and production of ACh by immune cells following type 2 immune challenge can promote host type 2 immune responses.^8^ Moreover, ACh-producing T cells can contribute to control of chronic viral infection,^9^ and CD4^+^ T-cell responses to ACh via the M3 muscarinic receptor are required for optimal adaptive immunity to helminth and bacterial infections.^5^ In the spleen, lymphocytes are major effectors of the cholinergic anti-inflammatory pathway through their synthesis and release of ACh, which downregulates inflammation. ILC3 responses to cholinergic stimulation can also contribute to the cholinergic anti-inflammatory pathway by regulating neutrophilia in sepsis models. However, whether ILC3s themselves may be an immune cell–derived source of ACh capable of regulating immunity has not previously been investigated.

To identify whether lung ILC3s may produce ACh during acute protease-induced inflammation, we challenged ChAT(BAC)-eGFP reporter mice with papain. In addition to increased choline acetyltransferase (ChAT) production by CD3^+^CD4^+^ cells (and a trend toward increased production by Lin^–^CD45^+^CD127^+^ICOS^+^ ILC2s), we identified an increase in the ChAT-expressing ILC3-enriched (Lin^–^CD45^+^CD127^+^ICOS^–^) population, but with no effect on the number of NK (CD3^–^DX5^+^) cells in the lungs of papain-challenged mice (Fig 2, *A*). Therefore, RoRγt-expressing ILC3s increase synthesis of ACh following papain challenge.

**Fig 2:**
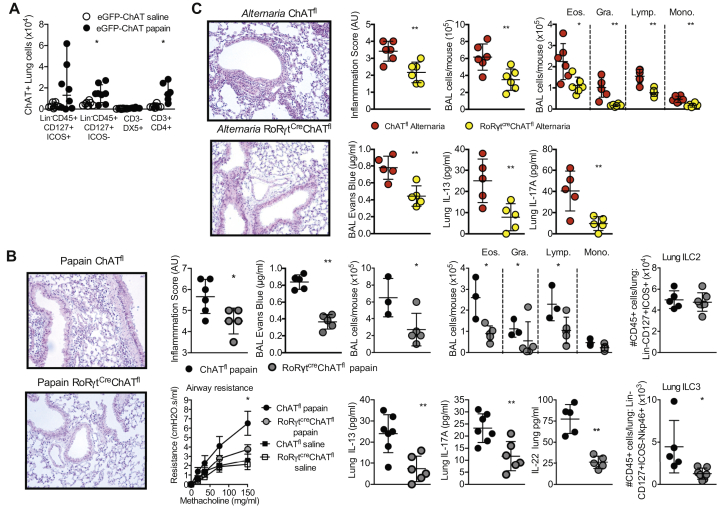
Disruption of ChAT in RoRγt^-^expressing cells is sufficient to protect against protease-induced lung inflammation. **A,** ChAT expression in pulmonary innate lymphoid cells (Lin^–^CD45^+^CD127^+^ICOS^+^, Lin^–^CD45^+^CD127^+^ICOS^–^, and CD3^–^DX5^+^ cells) or CD4 T cells (CD3^+^CD4^+^) in saline- or papain-challenged ChAT(BAC)–enhanced GFP (eGFP) reporter mice by flow cytometry. **B,** Representative hematoxylin and eosin staining of lung sections following papain challenge of ChAT^fl^ and RoRγt^Cre^ChAT^fl^ (disrupted ChAT expression driven by RoRγt-induced Cre expression) mice, pathology scoring and quantification of epithelial integrity by using EB. Airway resistance to methacholine challenge, quantification of airway cellular infiltration, detection of pulmonary IL-13 and IL-17A by ELISA, and detection of ILC2s (Lin^–^CD45^+^CD127^+^ICOS^+^) and NCR^+^ ILC3s (Lin^–^CD45^+^CD127^+^ICOS^-–^NKp46^+^) in the lung by flow cytometry in papain-challenged ChAT^fl^ and RoRγt^Cre^ChAT^fl^ mice. **C,** Representative hematoxylin and eosin staining of lung sections, pathology scoring, quantification of epithelial integrity by using EB, quantification of airway cellular infiltration, and detection of pulmonary IL-13, IL-22, and IL-17A by ELISA in *A alternata*–challenged ChAT^fl^ and RoRγt^Cre^ChAT^fl^ mice. Data are representative of 2 or 3 equivalent experiments; n = 3 to 9 mice/group. All data are a comparison of unchallenged and saline-treated or papain-challenged C57BL/6-background mice. Values represent means ± SDs; ∗*P* < .05; ∗∗*P* < .01; ∗∗∗*P* < .001. *AU*, Arbitrary units; *BAL*, bronchoalveolar lavage fluid; *Eos.*, eosinophil; *Gra.*, granulocyte; *Lymp*, lymphocyte; *Mono.*, monocyte.

To test whether ILC3 synthesis of ACh contributes to papain-driven pathology, we generated RoRγt^Cre^ChAT^loxp^ mice. These mice lack the ability to generate ACh following deletion of ChAT in RoRγt-expressing cells. Papain challenge of RoRγt^Cre^ChAT^loxp^ mice resulted in decreased histologic detection of inflammation and vascular leakage of EB in BALF compared with in the BALF of ChAT^loxp^ mice (Fig 2, *B*), along with reduced methacholine-induced airway resistance and reduced numbers of neutrophils and eosinophils in the BALF (Fig 2, *B*). Detection of cytokines in lung homogenates identified reduced IL-13 and IL-17A levels as well as reduced IL-22 levels, in RoRγt^Cre^ChAT^loxp^ mice as compared with in the controls (Fig 2, *B*). Therefore, disruption of ChAT in RoRγt^+^ cells was sufficient to recapitulate the reduced pathology seen in papain-challenged *Ror*c^–/–^ mice (Fig 1). Quantification of ILCs revealed equivalent ILC2 (Lin^–^CD45^+^CD127^+^ICOS^+^) numbers but decreased NCR^+^ ILC3 (Lin^–^CD45^+^CD127^+^ICOS^–^NKp46^+^) numbers between papain-challenged WT and RoRγt^Cre^ChAT^loxp^ mice (Fig 2, *B*).

An equivalent phenotype was demonstrated following *Alternaria alternata* extract–driven acute allergic inflammation (Fig 2, *C*). As with papain, *A alternata* extract challenge of RoRγt^Cre^ChAT^loxp^ mice resulted in decreased histologic detection of inflammation and vascular leakage of EB in BALF along with reduced methacholine-induced airway resistance, reduced numbers of neutrophils and eosinophils in the BALF, and reduced detection of IL-13 and IL-17A in lung homogenates compared with in ChAT^loxp^ mice (Fig 2, *C*).

In summary, following acute protease challenge, we found raised IL-13, IL-23, IL-22, and IL-17A expression in the lung and increased numbers of ILC3s. Papain challenge did not induce elevated CD4^+^ T-cell IL-17 levels irrespective of raised RoRγt^+^CD4^+^ T-cell numbers. Moreover, in the absence of T cells, papain-induced lung inflammation was maintained but abrogated when IL-23 or RoRγt function was disrupted. This strongly supports ILC3 expansion in the lung as a driving factor in IL-17–associated inflammation following an acute protease lung challenge. Reduction in airway cholinergic responsiveness led us to investigate whether ILC3s were a physiologically relevant source of ACh following papain challenge. We tested this by generating RoRγt^Cre^ChAT^loxp^ mice that lack the ability to generate ACh in RoRγt-expressing cells. Remarkably, this ILC3-biased disruption of ChAT expression protected against pathology to an extent equivalent to that seen in *Rorc*^–/–^ mice. This identifies RoRγt^+^ cell expression of ChAT as an important component in the promotion of protease-mediated allergic lung pathology. Indeed, these data support ILC3s expressing ACh as playing a central role in initiation of the IL-17–promoted allergic inflammatory cascade. These findings place ILC3 synthesis of ACh as a central requirement for allergic lung inflammation, adding a critical new paradigm to our understanding of cholinergic responses in driving allergic lung inflammation and pathology.

## Figures and Tables

**Fig E1:**
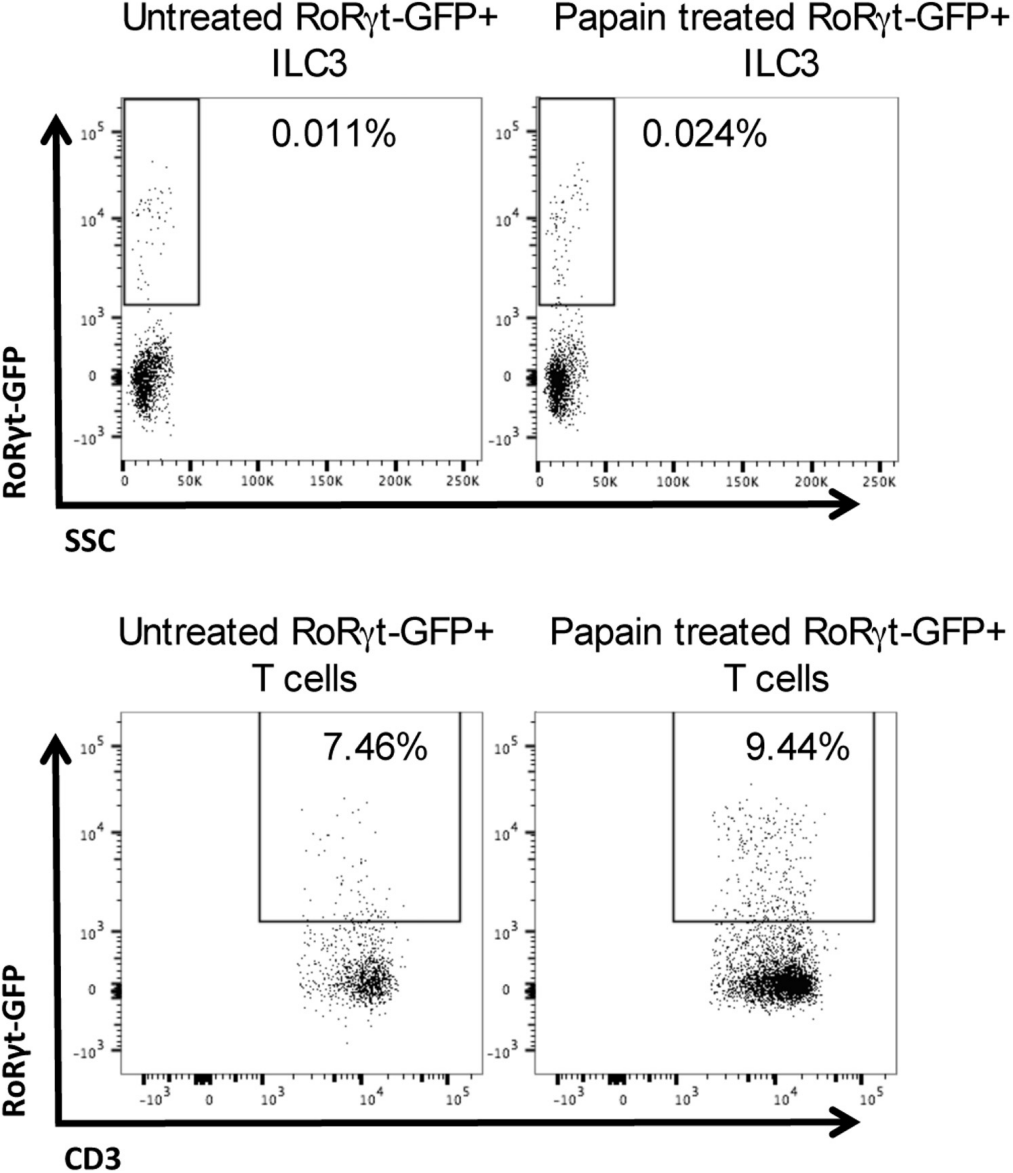
RoRγt-GFP show increased ILC3s and RoRγt^+^ T cells. ILC3s (Lin^*–*^CD45^+^CD127^+^ICOS^*–*^RoRγt-GFP^+^) and T_H_17 T cells (CD3^+^CD4^+^RoRγt-GFP^+^) were detected in the lung by flow cytometry using RoRγt-GFP reporter mice following papain challenge. *SSC*, Side scatter.

**Fig E2:**
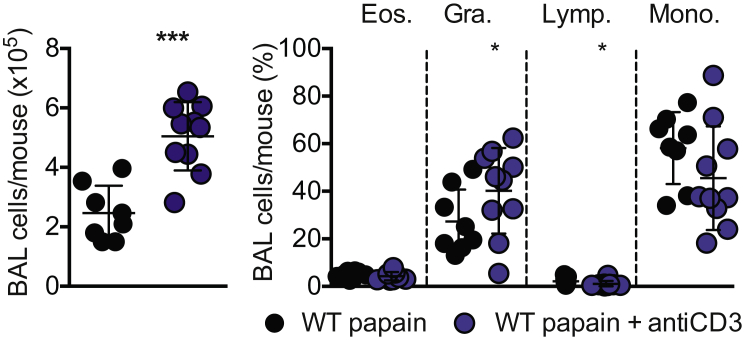
CD3-depleted ChAT-GFP mice do not show reduced inflammation to papain. Mice were treated daily with anti-CD3 antibody administered intraperitoneally 1 hour before papain challenge at a dose of 50 μg/mouse. BALF cellular infiltration was assessed 24 hours after final administration of papain. *BAL*, Bronchoalveolar lavage fluid; *Eos.*, eosinophil; *Gra.*, granulocyte; *Lymp*, lymphocyte; *Mono.*, monocyte.

**Fig E3:**
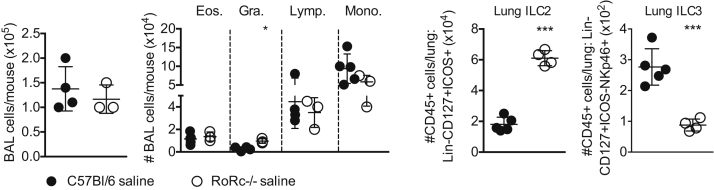
*Rorc*^*–/–*^ mice do not show significant baseline differences from C57Bl/6 mice in terms of BALF cell composition. BALF immune cell infiltration and levels of lung ILC2s (Lin^*–*^CD45^+^CD127^+^ICOS^+^) and NCR^+^ ILC3s (Lin^*–*^CD45^+^CD127^+^ICOS^*–*^NKp46^+^) in saline-treated C57Bl/6 and *Rorc*^*–/–*^ mice were analyzed by flow cytometry. *BAL*, Bronchoalveolar lavage fluid; *Eos.*, eosinophil; *Gra.*, granulocyte; *Lymp*, lymphocyte; *Mono.*, monocyte.

## Methods

### Mice used and animal procedures

All mice used in this study were 6- to 12-week-old C57BL/6 background mice. The mouse strains used were C57BL/6, *RAG2*^–/–^, ChAT(BAC)-eGFP,^E1^ RoRγt-eGFP,^E2^ RoRγt-KO (*Rorc*^–/–^),E3, E4 RoRγt^Cre^,^E3^ and ChAT^loxp^.^E5^ *Rorc*^–/–^ mice are deficient in expression of transcript variant 2 of *RORC*, encoding RoRγt. RoRγt is necessary for the development of lymph nodes and Peyer patches, which fail to develop in *Rorc*^–/–^ mice.^E4^ RoRγt is expressed by many cells, including immature double-positive (CD4^+^CD8^+^) αβ thymocytes, RoRγt^+^ T_H_17 T cells, and ILC3s, which comprise NCR^+^ ILC3s and NCR^–^ ILC3s, as well as CCR6^+^ lymphoid tissue inducer cells. RoRγt^Cre^ and ChAT^loxp^ mice were crossed over 5 generations to generate RoRγt^Cre^ChAT^fl^ mice. The ChAT(BAC)-eGFP mouse has been demonstrated to faithfully report ChAT expression and ACh synthesis capacity across a number of hematopoietic immune cell types.^E1^ As the major rate-limiting enzyme of crucial significance for ACh synthesis, demonstration of ChAT expression by a specific cell type is widely considered to reflect the cholinergic-synthesizing nature of the cell.

The mice were anesthetized by isoflurane followed by intranasal administration of 25 μg of papain (Calbiochem, Darmstadt, Germany) in 40 μL of saline solution per mouse once per day on days 1, 2, and 3. On day 4 mice, were humanely killed by CO_2_ inhalation 24 hours after the final administration of papain. *A alternata* extract was administered daily for 3 days at a dose of 25 μg per mouse by the endotracheal route under light isoflurane anaesthesia, before the mice were humanely killed on day 4 by CO_2_ inhalation. Anti–IL-23 antibody or isotype control (Bio X cell, Lebanon, NH; BR0313 or rat IgG1) was given daily 1 hour before papain challenge at a dose of 200 μg per mouse via the endotracheal route. Anti-CD3 (Biolegend, San Diego, Calif; catalog no. BL100208) antibody or isotype controls was given via intraperitoneal injection daily 1 hour before papain challenge at a rate of 50 μg per mouse. After the mice were humanely killed, BALF was collected before cardiac perfusion with ISOTON II (acid-free balanced electrolyte solution, Beckman Coulter, Krefeld, Germany), after which the lungs were collected and sampled for analyses.

All of the animal experimental protocols complied with French ethical and animal experiments regulations (see Charte Nationale, Code Rural R 214-122, 214-124 and European Union Directive 86/609/EEC) and were approved by the Ethics Committee for Animal Experimentation of CNRS Campus Orleans, registered (No. 3) by the French National Committee of Ethical Reflexion for Animal Experimentation (CLE CNRS Campus Orleans 2013-1006). All of the South African experiments were carried out in accordance with South African Veterinary Council regulations and were approved by the University of Cape Town Faculty of Health Sciences Animal Ethics Committee.

### Histology

The left lobe of lung was fixed in 4% buffered formaldehyde and paraffin embedded under standard conditions. Tissue sections (3-μm) were stained with standard hematoxylin and eosin and periodic acid–Schiff. The histologic score of the pathology was determined by a semiquantitative assessment on a scale of 0 to 5 for cell infiltration (with increasing extent). The slides were blindly examined by 2 investigators with a Leica microscope (Leica, Solms, Germany).

### Quantification of protein, EB, and cellular infiltration in BALF

Bronchoalveolar lavage was performed by 4 lavages of lung with 500 μL of saline solution via a cannula introduced into the mouse trachea. BALF samples were centrifuged at 400 *g* for 10 minutes at 4°C, the supernatants were stored at –20°C for analysis, and pellets were recovered to prepare Cytospin (Thermo Scientific, Waltham, Mass) on glass slides followed by staining with Diff-Quik stain (Merz & Dade AG, Dudingen, Switzerland). Differential cell counts were performed with at least 300 cells. Vascular leakage was quantified by protein and EB concentration in the BALF. EB in BALF was measured 45 minutes after intravenous injection of 0.3% EB; the measurement was performed by absorbance at 460 nm, as described elsewhere.^E6^ Extravasation was expressed as micrograms of EB per milliliter (μg of EB/mL) of BALF supernatant.

### Flow cytometry

Lungs were digested in RPMI 1640 medium containing 100 U/mL of penicillin, 100 U/mL of streptomycin, 1 mg/mL of DNase I (Sigma, St Louis, Mo), and 125 μg of liberase (Roche, Basel, Switzerland) for 1 hour at 37°C under rotation. After digestion, RPMI 1640 medium supplemented with 10% FCS was added. Cells were dissociated by passage through a 70-μm cell strainer and centrifuged at 400 *g* for 5 minutes at 4°C. Pellets were resuspended in red blood cell lysis buffer (Stem Cell Technologies, Vancouver, British Columbia, Canada) and incubated for 10 minutes on ice. Lysis was stopped by addition of RPMI 1640 medium and centrifuged again at 400 *g* for 5 minutes at 4°C. Pellets were resuspended in RPMI 1640 medium supplemented with 10% FCS and passed through a 40-μm cell strainer. ILC2s were identified by using a Lin cocktail, CD45, CD127, and ICOS panel. ILC3s were identified by using a panel containing a Lin cocktail, CD45, CD127, ICOS, and NKp46 or RoRγt, as indicated. Cytokine-expressing T cells were identified by using a panel containing CD3, CD4, IL-5, IL-13, or IL-17, as indicated. NK cells were identified by using a panel containing CD3 and DX5. All FACS antibodies were from Biolegend.

### ELISA

Homogenized lungs were tested for IL-13 and IL-17 by using commercial ELISA kits (eBiosciences, San Diego, Calif) according to the manufacturer's instructions.

### Determination of bronchial hyperresponsiveness

For invasive measurement of dynamic resistance, mice were anesthetized by intraperitoneal injection of solution containing ketamine (100 mg/kg, Merial, Duluth, Ga) and xylazine (10 mg/kg, Bayer, Leverkusen, Germany), paralyzed by using D-tubocuranine (0.125%, Sigma), and intubated with an 18-gauge catheter. Respiratory frequency was set at 140 breaths per minute with a tidal volume of 0.2 mL and a positive end-expiratory pressure of 2 mL of H_2_O. Increasing concentrations of aerosolized methacholine (9.375, 18.75, 37.5, 75, and 150 mg/mL) were administered. Resistance was recorded by using an invasive plethysmograph (Buxco, London, United Kingdom). Baseline resistance was restored before administration of the subsequent doses of methacholine.^E7^

### Statistical analysis

Data were analyzed by using Prism software, version 5 or 6 (GraphPad Software, San Diego, Calif). Either a Mann-Whitney *t* test or the parametric 1-way ANOVA test with Bonferroni multiple-comparison was used to assess significance. Values are expressed as means ± SDs.
